# Case Report: From Misdiagnosis to Accurate Identification: Managing a Case Series of *Trichophyton rubrum* Infections

**DOI:** 10.3390/microorganisms13040895

**Published:** 2025-04-13

**Authors:** Vivian Tullio, Michele Panzone, Ornella Cervetti, Janira Roana, Narcisa Mandras

**Affiliations:** 1 Department Public Health and Pediatrics, Microbiology Division, University of Turin, 10126 Torino, Italy; janira.roana@unito.it (J.R.); narcisa.mandras@unito.it (N.M.); 2 A.O.U Città della Salute e della Scienza, San Lazzaro Hospital, 10126 Turin, Italy; mpanzone@cittadellasalute.to.it; 3 Department Medical Sciences, University of Turin, 10126 Torino, Italy; ornella.cervetti@unito.it

**Keywords:** widespread dermatophytosis, *Trichophyton rubrum*, correct diagnosis, appropriate therapy

## Abstract

In recent decades, despite being well-known, dermatophytosis has seen a resurgence and an increase in the incidence of infections, with dermatophytes such as *Trichophyton rubrum* being the most common agents. Dermatophytosis pathogenesis involves complex interactions between the host, agent, and environment. In many cases, dermatophytosis can be mistaken for other pathologies, which leads to incorrect therapies and the consequent non-recovery of the patient. In this paper, we describe five previously undiagnosed cases of diffuse *T. rubrum* dermatophytosis because they represent the clinical manifestations that affect several sites at the same time and that, if not properly diagnosed and treated, can lead to severe, widespread, chronic, and difficult-to-treat dermatophytosis. This case series of five instances of misdiagnosed *T. rubrum* dermatophytosis was later accurately diagnosed and successfully treated with systemic terbinafine hydrochloride 250 mg/die for at least four weeks up to twelve or sixteen, and topical azoles (sertaconazole nitrate 2%) as well. This case series highlights the need to make an accurate diagnosis and avoid misidentifications while offering insightful information about the clinical presentation and treatment of these illnesses.

## 1. Introduction

The skin protects the fundamental tissues and plays a key role as a first-line defense framework against outside natural changes and invading pathogens. It is colonized by a complex microbiome that includes bacteria, fungi, and even bacteriophages, some of which could cause disease in certain situations [1,2,3]. Among fungi, dermatophytes invade body-keratinized tissues such skin, hair, and nails, causing an infection known as dermatophytosis that affects 20–25 percent of the world’s population [4,5,6]. In recent decades, despite being well-known, dermatophytoses have seen a resurgence and an increase in the incidence of infections in some regions. This trend can be attributed to various factors, including increased urbanization, higher population density, changes in lifestyle, and hygiene practices [7]. The development of resistance to commonly used antifungal treatments among dermatophytes has been increasingly documented [8]. This resistance makes infections harder to treat and can lead to more widespread and persistent infections, posing significant public health challenges [9,10].

Global travel and migration have facilitated the spread of dermatophytes to new regions, introducing new strains and raising infection rates [11]. Changes in climate, particularly global warming, have created more favorable conditions for the growth of these fungi [12]. Historically, dermatophytes were classified into three anamorphic genera—*Microsporum*, *Trichophyton*, and *Epidermophyton*—with around 40 species. Recent taxonomic revisions have expanded this classification to eight genera, including *Arthroderma*, *Lophophyton*, and *Nannizzia* [13]. These fungi can cause injury at different body sites, clinically classified as *tinea*: *tinea capitis* (head), *tinea corporis* (body), *tinea cruris* (groin), *tinea pedis* (feet), and *tinea unguium* (nails) [3]. The pathogenesis of dermatophytosis involves the complex interaction between the host, agent, and the environment [14,15]. The factors which predispose a person to this infection are older age and underlying diseases (i.e., diabetes mellitus, obesity, lymphomas, immunocompromised status, psoriasis, Cushing’s syndrome, HIV, etc.), which could produce severe, widespread, or difficult-to-treat dermatophytosis [14,15]. Some body areas are more susceptible to dermatophytosis infection development, such as intertriginous areas where excess sweating, maceration, and alkaline pH favor the dermatophytes growth [3]. Agent characteristics are also important in infection development. In fact, the components of the fungal cell wall, including mannans, are crucial in the down-modulation of the cell-mediated immune host response [16]. Furthermore, patients with various lesions on the body’s surface are often misdiagnosed and treated with ineffective therapies, thus leading to relapses and treatment failure [15,17,18]. As a result, the diagnosis of dermatophytosis with diffuse lesions must be based on a correct medical history and microbiological research that includes the correct identification of the isolated fungal etiological agents. One of the most widespread dermatophytes in the world is *T. rubrum*, an agent of most cases of diffuse, recurrent, and chronic dermatophytosis, along with *T. mentagrophytes* [3].

To better understand the clinical and mycological aspects of *T. rubrum* dermatophytosis and improve diagnosis and treatment, we present five examples of previously misdiagnosed cases of the disease in patients with diffuse body lesions who were admitted to the University of Torino’s Medical Science Department at the San Lazzaro Hospital (Turin, Italy). In addition to providing useful information regarding the clinical presentation and management of these disorders, this case series emphasizes the need to accurately diagnose patients and prevent misidentifications.

## 2. Case Presentation Section: Patients and Therapy

### 2.1. First Patient

**Figure 1:**
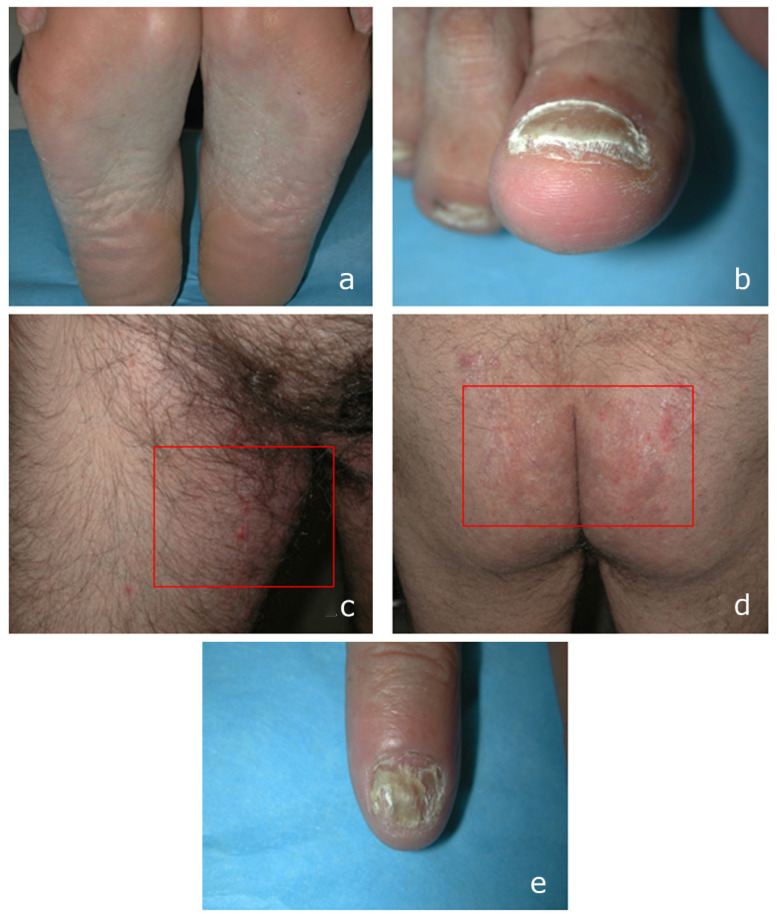
A 48-year-old, male, Caucasian: (**a**) erythematous and squamous lesions on the soles; (**b**) toenails plate; (**c**) inguinal area and (**d**) buttocks (erythematous and scaling lesions); and (**e**) hyperkeratosis and distal onycholysis on fingernail.

*Clinical background and findings*. The first patient, a 48-year-old Caucasian male, only reported erythematous and desquamative lesions on the soles of his feet, which had persisted for a minimum of 8 months (Figure 1a). Prior evaluations by a general practitioner in other facilities led to a diagnosis of dyshidrosis of the feet, for which he was treated with a steroid cream; however, this treatment proved ineffective. Additionally, localized lesions had developed on the toenail plates (Figure 1b) over the past four weeks. These new manifestations prompted the patient to seek consultation at the dermatology department of San Lazzaro Hospital. Regarding the present and following a closer inspection, the groin–crural site (Figure 1c), as well as the buttocks (Figure 1d), had erythematous and scaling lesions. Hyperkeratosis and distal onycholysis were detected on the fingernail plate (Figure 1e). The referral did not reveal any underlying pathologies. Dermatologists assumed a diffuse mycosis, in particular, dermatophytosis, and proposed a therapy with topical azoles (sertaconazole nitrate cream 2%, two applications/day) on the area of the affected skin and on the immediately adjacent area, and systemic terbinafine hydrochloride 250 mg/die. A short-term follow-up was 12 and 24 weeks. This new therapy allowed the clinical healing of all lesions and negative culture findings after 12 weeks.

### 2.2. Second Patient

**Figure 2:**
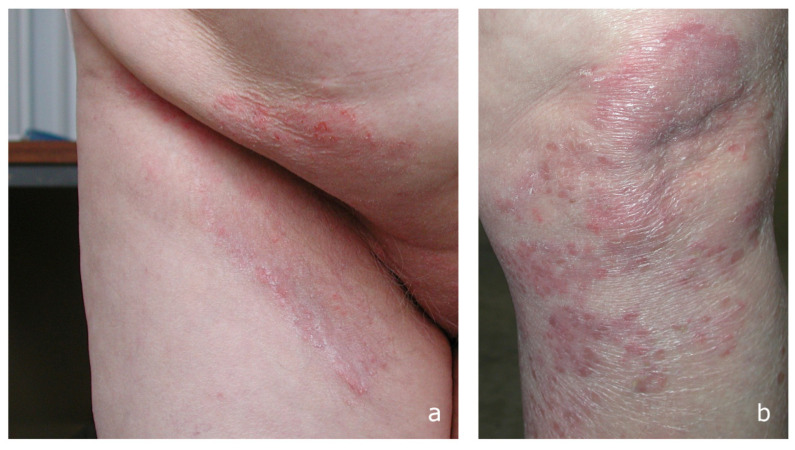
A 78-year-old, female, Caucasian: (**a**) extensive erythema with papules at the opening of hair follicles in the inguinal region; and (**b**) left knee with flaking in net margins.

*Clinical background and findings*. The second patient was a Caucasian female, 78 years old. For the past two weeks, she has been complaining of desquamative and erythematous lesions in the inguinal area, previously diagnosed, in other facilities, as contact dermatitis and treated with emollient cream without success. On the current physical examination, damage to hair follicles in the groin was observed with whitish papulo-pustular lesions, evidence of epidermal parasitization (Figure 2a). Both the buttocks and the thighs showed extended erythema. Scales were also seen between the clean borders of the right leg (Figure 2b) and left knee. Furthermore, hyperkeratosis affected the left foot’s sole and toenails, making the nail plate thicker, friable, and yellowish. There have been no reports of underlying disease problems. Based on additional lesions observed on the body, which had not previously been considered, dermatologists assumed a diffuse dermatophytosis. The patient was successfully treated with topical sertaconazole and systemic terbinafine hydrochloride 250 mg/die for 6 weeks. A short-term follow-up was 3 and 6 weeks, and 12 months. Lesions of the skin were fully recovered and culture findings were negative. Both direct mycological and culture assays for nails were negative after four months.

### 2.3. Third Patient

**Figure 3:**
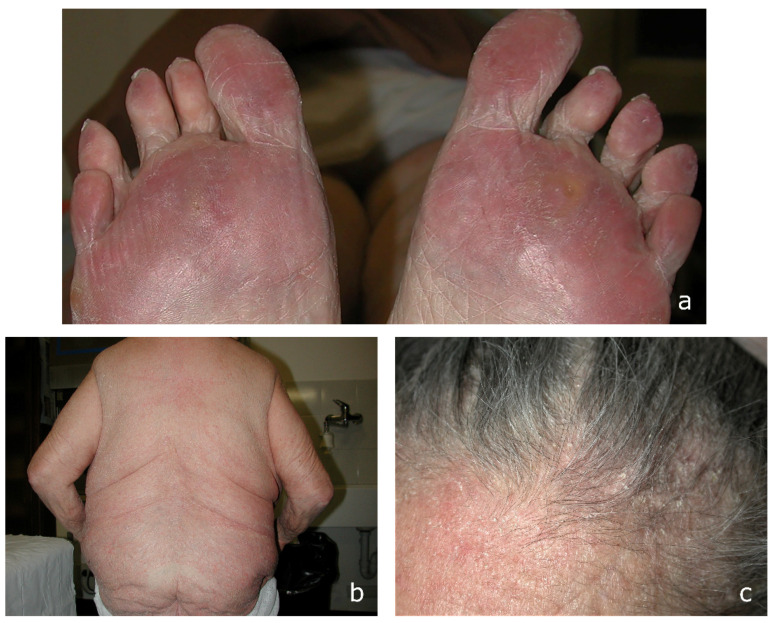
A 69-year-old, female, Caucasian, with rheumatoid arthritis: (**a**) sole and toenail hyperkeratosis; (**b**) extensive erythema on lower back area diagnosed as psoriasis; and (**c**) scalp with flaking dandruff and thinning hair.

*Clinical background and findings*. For the past 20 years, a 69-year-old Caucasian woman was treated for rheumatoid arthritis by other physicians with methotrexate (7.5 mg/week) and prednisone (5 mg/die) cycles. Since 2006, the patient has been treated unsuccessfully with an emollient lotion and topical medications for superficial psoriasis. At Dermatology San Lazzaro Hospital, the current physical examination of the skin revealed the presence of *tinea pedis* characterized by plantar and toenail hyperkeratosis (Figure 3a). Both the back and left palm showed squamous lesions but not inflammation. The erythematous–desquamative lesions were extended to the lumbar back area (Figure 3b), and the back of the leg, assuming a reniform appearance. Damage to the scalp was also noted, with scaly dandruff and scant hair. (Figure 3c). Based on additional lesions observed on the body, which had not previously been considered, dermatologists assumed a diffuse dermatophytosis. Therapy involved the use of topical sertaconazole and systemic terbinafine hydrochloride (250 mg/die). A short-term follow-up was 3, 4, and 6 weeks, 5 and 12 months. After 4 weeks of therapy, the regression of skin lesions in the lumbar back area and back of the leg posterior was observed. After five months, the culture results for the nails and scalp were negative.

### 2.4. Fourth Patient

**Figure 4:**
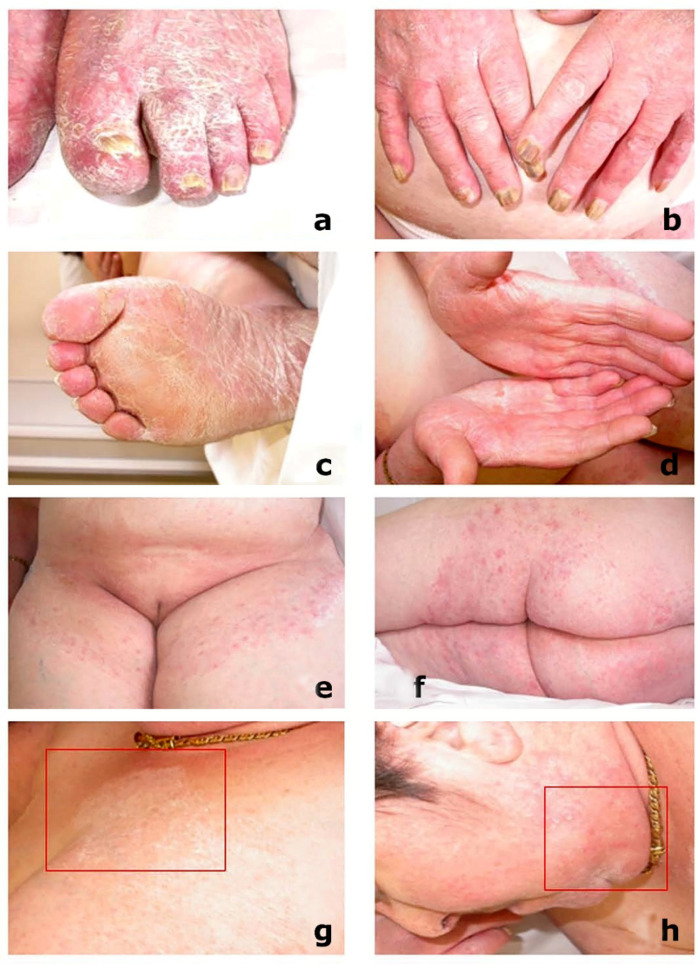
A 68-year-old, female, Caucasian, with rheumatoid arthritis: (**a**,**b**) lamellar toenails and fingernails desquamation; (**c**,**d**) sole and palms hyperkeratosis; (**e**) scaling lesions in the abdomen and inguinal area; (**f**) buttocks and thighs; (**g**) neck; and (**h**) chin previously diagnosed as psoriasis (scaling lesions).

*Clinical background and findings*. A 68-year-old Caucasian female complained of chronic erythematous scaly lesions on her arms and legs, abdomen, chest, and face, which were previously considered in other facilities as diffuse psoriasis and treated with a softening cream without improvement. Rheumatoid arthritis was referred as the underlying pathology, which was managed for a number of years with prednisone (25 mg/die). At Dermatology San Lazzaro Hospital, on the current dermatological examination, the patient showed severe lamellar desquamation of the toenails/fingernails, as well as sole and palm hyperkeratosis (Figure 4a–d). In addition, scaling lesions with cutting edges were found in the crural–groin area (Figure 4e), buttocks (Figure 4f), neck (Figure 4g), and chin (Figure 4h). Based on additional lesions observed on the body, which had not previously been considered, dermatologists assumed a diffuse dermatophytosis. All lesions were successful treated with topical sertaconazole and systemic terbinafine hydrochloride (250 mg/die). A short-term follow-up was 3, 4, 6, and 16 weeks, and 5 and 12 months. After 16 weeks, the culture findings were negative.

### 2.5. Fifth Patient

**Figure 5:**
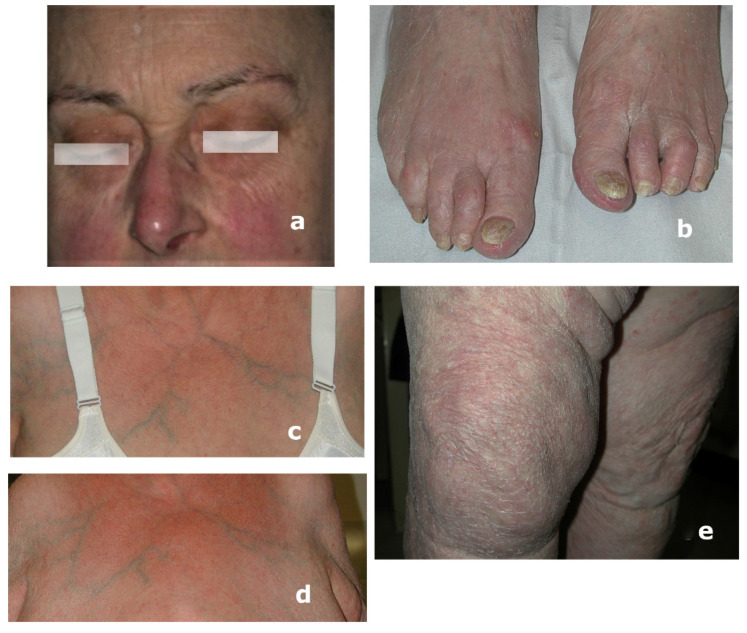
A 74-year-old, female, Caucasian: (**a**) face erythematous lesions previously diagnosed as contact dermatitis; (**b**) distal onycholysis on the toenails and signs of desquamation on the dorsum of the foot; (**c**,**d**) neckline and back extended erythema; and (**e**) leg scaling lesions.

*Clinical background and findings.* The fifth patient, a Caucasian female, 74 years old, was referred for erythematous lesions on the face for at least 6 weeks. These lesions were previously diagnosed by other physicians as contact dermatitis and treated with emollient cream without success (Figure 5a). Lesions in other parts of the body had not been searched for and/or examined. The current closer examination revealed hyperkeratosis and distal onycholysis on toenail plate, as well as signs of desquamation on the dorsum of the foot (Figure 5b). Both the neckline and the back showed extended erythema (Figure 5c,d). The erythematous-extended desquamative lesions were also detected on both legs (Figure 5e). No underlying pathologies were referred. Based on additional lesions observed on the body, dermatologists assumed a diffuse dermatophytosis. The proposed therapy with topical azoles (sertaconazole nitrate cream 2%, two applications/day) on the area of affected skin and systemic terbinafine hydrochloride 250 mg/die, which allowed for the clinical healing of all lesions and negative culture findings after 10 weeks. A short-term follow-up was 2, 5, and 10 weeks, and 3 and 12 months. Both direct mycological and culture assays for nails were negative after three months.

## 3. Mycological Study and Analysis

For all the patients described, once they arrive at the San Lazzaro Hospital, the samples were collected from all skin and nail lesions at the Medical Science Department, University of Torino, during routine diagnostic activities. Informed written consent was collected from all patients before the procedure. To detect fungal components, direct observation was performed with a 20% KOH solution.

**Figure 6:**
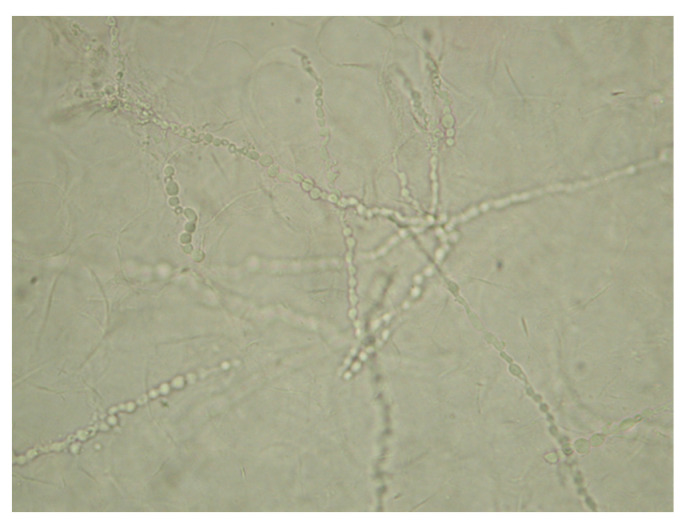
20% KOH direct microscopic examination (400×). Dermatophyte-typical septate hyphae.

A direct microscopic examination of the samples revealed typical septate hyphae of dermatophytes (Figure 6).

Therapy was started immediately if the KOH examination indicated the presence of fungi in the sample.

The samples were then cultured on Sabouraud dextrose agar and Mycobiotic agar with chloramphenicol (0.05 g/L) and cycloheximide (0.4 g/L) (Merck, KGAA, Darmstadt, Germany) at 25 °C for at least 3 weeks and examined weekly to detect dermatophytes.

**Figure 7:**
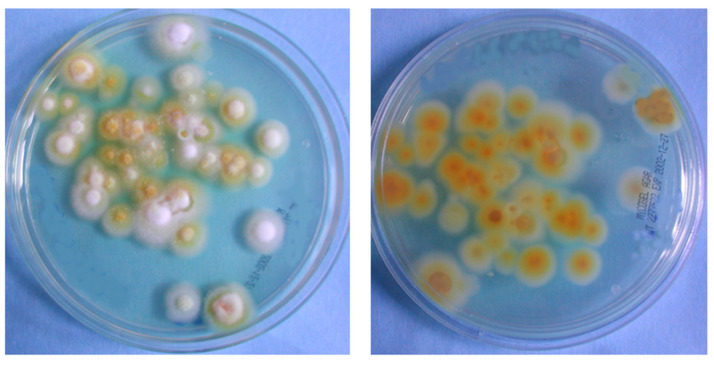
*T. rubrum* colonies. Downy white-colored colonies with brownish-yellow reverse pigment (Patients 1, 2, and 5).

**Figure 8:**
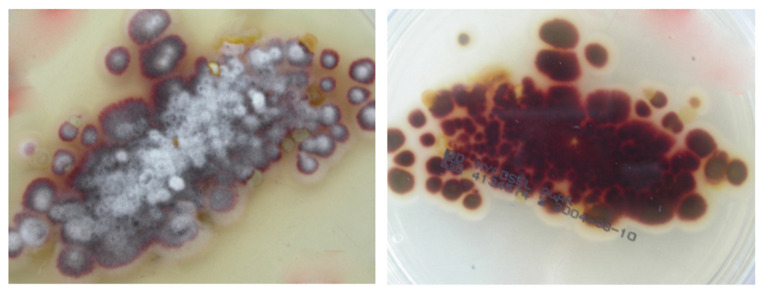
*T. rubrum* colonies. Downy white-colored colonies with a deep wine-red reverse pigment (Patients 3, 4).

Fungi were initially identified using a macroscopic and microscopic examination of colonies and asexual reproductive structures at the Bacteriology and Mycology Laboratory, Department of Public Health and Pediatrics, Microbiology Division, University of Torino (Italy) [19]. On a microscopic examination of the colonies, there were a few teardrop-shaped microconidia along the septate hyphae. Incubation on plates resulted in downy white colonies with a brownish-yellow (Figure 7; Patients 1, 2, and 5) or deep wine-red reverse pigment (Figure 8; Patients 3, 4). On the basis of the macroscopic and microscopic morphological characteristics of the fungal isolates, the strains were classified as two forms of *T. rubrum* in all lesions (Figure 7 and Figure 8).

*T. rubrum* strains’ identification was confirmed by the use of molecular techniques at the Dept. of Life Sciences and Systems Biology, University of Torino (Italy), to lead to a correct species diagnosis [20]. Briefly, fungal DNA was extracted from a fresh dermatophyte colony using the NucleoSpin kit (Macherey Nagel GmbH, Duren, DE, USA), following the manufacturer’s instructions. Using the primers ITS1 (5′-TCCGTAGGTGAACCTGCGG-3′) and ITS4 (5′-TCCTCCGCTTATTGATATGC-3′), all isolates were identified to the species level by sequencing the internal transcribed spacers (ITS) 1 and 2 regions, as well as the 5.8S ribosomal DNA subunit. PCR reactions were performed in 50 μL final volumes and consisted of 0.5 μL Taq DNA Polymerase (Qiagen 5 U/μL), 5 μL PCR Buffer (10×), 2.5 μL dNTP Mixture (dATP, dCTP, dGTP, dTTP; 10 mM), 2 μL MgCl_2_ (25 mM), 2.5 μL of each primer (10 μM), 1 μL genomic DNA extract, and 35.5 μL distilled-deionised water. The steps for the polymerase chain reaction (PCR) conditions were as follows: an initial denaturation at 95 °C for 5 min, 35 denaturation cycles at 95 °C for 40 s, annealing at 55 °C for 50 s, an extension at 72 °C for 50 s, and a final extension at 72 °C for 8 min. PCR products were visualized under UV light (BIO-RAD Universal Hood II) on 1.5% agarose electrophoresis gels stained with ethidium bromide. The Macrogen, Inc. (Seoul, Republic of Korea) Europe Lab carried out the purification and sequencing of PCR products [20]. ITS sequences were subjected to Basic Local Alignment Search Tool (BLAST) searches in the GenBank NCBI database (https://blast.ncbi.nlm.nih.gov/Blast.cgi). Sequence-based species identification was defined by ≥99% sequence similarity.

Moreover, fungal identification was completed to the species level and reported using Microflex LT MALDI-TOF (Bruker Daltonics GmbH & Co. KG, Bremen, Germany), which is now a routine assay in many hospital microbiological laboratories.

It is worth pointing out that molecular identification and the MALDI-TOF technique were used only to validate the macroscopic and microscopic identification of colonies.

In all five cases, *T. rubrum* was always isolated at the foot level and the clinical signs of *tinea pedis* and *tinea unguium* were always present. Apart from the foot, the inguinal site, buttocks, palms, and fingernails were the most frequently examined anatomical areas (Table 1).

According to the information provided by the patients, the initial lesion was always located in the feet. Secondary lesions that had spread to other parts of the body were the most common reason for seeking medical attention. All of the patients had dermatophytosis, as well as secondary lesions caused by the same fungus in areas other than the initial lesion (Table 1). Patients 3 and 4 were those with the largest number of sites affected and those suffering from rheumatoid arthritis.

## 4. Discussion

Studies of the prevalence of dermatophytosis, found in about 20–25% of the world human population, indicate that the isolates most frequently observed in clinical samples belong to the species *T. rubrum* [4,14]. There are many cases reported in the literature ranging from mild to severe [5,21,22]. However, the case series reported in this paper highlights the importance and necessity of a correct and prompt diagnosis. In fact, failing to distinguish mycotic lesions, particularly those caused by dermatophytes, from other pathologies leads to an incorrect diagnosis with the consequent inadequate therapy and worsening of symptoms. Establishing an accurate diagnosis is essential for effective treatment and can help prevent antifungal resistance [23]. Our cases, even if few, reveal that previous medical visits had not highlighted the presence of dermatophytosis, both probably because the patient, despite having lesions throughout the body, had not reported all the lesions present, and also because the physician had not investigated the other lesions, or also because mycological examinations had not been carried out on all the lesions present. These circumstances can result in mycosis recurrence and spread to other parts of the body [24].

*T. rubrum* infection is generally restricted and poorly inflammatory, but it can spread and affect the entire integument, probably due to immune response suppression, in some groups of people, such as immunocompromised people, such as those suffering from AIDS, those undergoing chemotherapy, or the elderly or patients with concomitant diseases, according to our cases [25]. According to the literature [26,27], immunocompetent patients can also develop diffuse chronic dermatophytosis, which is most commonly linked with *tinea pedis*, *tinea manuum*, and *tinea unguium*.

Kick and Korting (2001) defined as *T. rubrum* syndrome the clinical manifestations that simultaneously affect at least four localizations, including the hands, feet, nails, thighs, abdomen, armpits, and groin [28]. Based on a remote pathological history, the clinical cases reported in this study fall into this category. In fact, *T. rubrum* was isolated from all of the patients’ samples taken at different body sites (Table 1). The initial lesion appeared on the sole/dorsum of the foot, causing *tinea pedis*. This evidence is consistent with those seen in the literature [17,29], indicating that this is the major infection location. In addition to *tinea pedis*, *tinea unguium* was also present in all subjects at the same time. This progressive expansion up to the toenails, the second site always involved, appears to have served as a reservoir for the infection, which then spread to other parts of the body, such as the legs, groin, hands, face, and scalp.

According to the epidemiological studies by Szepietowski et al. [30] and Kawai et al. [31], there is a recurrent relationship between *tinea pedis* and *tinea unguium* of the toenails. It should be underlined that therapy for *tinea pedis* and *tinea unguium* is unquestionably critical in preventing the spread of the fungal infection from a localized to widespread illness. In fact, *tinea unguium* is more resistant to therapy and, despite adequate treatment [32], eradication is difficult, if not impossible. In immunocompetent individuals, failure to respond to therapeutic treatment can be caused by a variety of circumstances, including poor patient compliance, failure to complete prescribed medication, drug metabolism in the liver, drug interactions, problems penetrating the stratum corneum of the skin, and drug resistance.

*T. rubrum* dermatophytosis has been shown to cause clinical diagnostic issues in the past. The diagnostic certainty, which must be based on a correct medical history and an adequate microbiological study that includes the identification of the isolated fungal species, becomes essential for the therapeutic treatment and eradication of the infection [33,34]. If the following pathological history does not contain a thorough examination of the patient, but merely the observation of a single lesion, the treatment decision may be inadequate or incorrect. In fact, it can happen, as in the fifth clinical case studied, that the family doctor to whom the subject turns bases his therapeutic choices only on the observation of a single lesion. In this patient, the lesions on the face did not present the typical clinical features of a *T. rubrum* infection, which directed the doctor towards contact dermatitis and the prescription of an emollient cream. The patient did not respond to treatment and, therefore, was advised to visit a dermatologist. The specialist conducted a complete physical examination of the body that allowed him to establish the fungal etiology of the infection. A similar situation appeared for patient 2, who was treated unsuccessfully with an emollient cream for contact dermatitis.

There are also cases of a specific type of glabrous skin dermatophytosis that has been altered in appearance because of the incorrect use of corticosteroids, either topically or systemically, for the treatment of other conditions. Patients 3 and 4 are included in this category. For rheumatoid arthritis and psoriasis, both patients were given oral or topical corticosteroids, respectively. In these circumstances, *tinea* tends to become chronic and to extend, mimicking a variety of skin diseases such as psoriasis, rosacea, systemic lupus erythematosus, and other forms of eczema [3,35]. In these circumstances, a mycological analysis is essential and should always be performed on any questionable or “abnormal” dermatological condition, especially in the presence of asymmetrical, dermatitis-like lesions usually with a symmetrical extension, such as psoriasis or eczema.

Topical and systemic therapies are required for extensive infections. Because the proper therapeutic treatment was not previously given or was given incorrectly in our group of patients, the infection gradually spread to several sites in the body. In some circumstances, oral terbinafine and azole formulations, such as itraconazole or fluconazole, may be beneficial [36]. However, as previously observed, the isolated *T. rubrum* strains were not very sensitive in vitro to the most common drugs used in therapy, such as itraconazole and/or ketoconazole [37]. Fortunately, our cases were treated with sertaconazole to avoid any resistance problems. In fact, following a period of treatment, all patients treated with an oral formulation of terbinafine in combination with a topical formulation of sertaconazole had healed clinical lesions, and culture tests were negative after a period of treatment that varied according to the severity and extent of the lesions.

## 5. Conclusions

The clinical diagnosis of *T. rubrum* dermatophytosis, in full-blown cases, does not present particular difficulties for dermatologists or medical specialists. However, in some instances, the presentation may be atypical or altered due to prior inappropriate treatments, making diagnosis more challenging. Since all the clinical cases discussed have *Tinea unguium* of the feet, it was crucial that we examine the nails for signs of fungal infection. Nail alterations are a strong indicator of a fungal etiology and may also point to underlying skin lesions caused by dermatophytes, especially in chronic and atypical forms. In such cases, when the skin involvement is extended to multiple sites, a multidisciplinary approach may be required, where an accurate clinical examination combined with a laboratory investigation becomes essential for an accurate diagnosis and for providing very useful information with which to initiate adequate therapy, which, in the case of the syndrome, must be of a systemic type and not only topical.

## Figures and Tables

**Table 1:** Data concerning the infected body site, the results of the corresponding direct microscopic analysis (20% KOH), and the fungal species identified.

	KOH Fungi		KOH Fungi		KOH Fungi		KOH Fungi		KOH Fungi	
Body Site	1st Patient		2nd Patient		3rd Patient		4th Patient		5th Patient	
**Soles/dorsum**	**+**	*T. rubrum*	**+**	*T. rubrum*	**+**	*T. rubrum*	**+**	*T. rubrum*	**+**	*T. rubrum*
**Toenails**	**+**	*T. rubrum*	**+**	*T. rubrum*	**+**	*T. rubrum*	**+**	*T. rubrum*	**+**	*T. rubrum*
**Legs/knee**		--	+	*T. rubrum*	+	*T. rubrum*	+	*T. rubrum*	+	*T. rubrum*
**Inguinal site**	**+**	*T. rubrum*	+	*T. rubrum*		--	+	*T. rubrum*		--
**Buttocks/thighs**	**+**	*T. rubrum*	+	*T. rubrum*		--	+	*T. rubrum*		--
**Palms**		--		--	+	*T. rubrum*	+	*T. rubrum*		--
**Fingernails**	**+**	*T. rubrum*		--		--	+	*T. rubrum*		--
**Face**		--		--		--	+	*T. rubrum*	+	*T. rubrum*
**Scalp**		--		--	+	*T. rubrum*		--		--
**Neckline/Abdomen/Back**		--		--		--	+	*T. rubrum*	+	*T. rubrum*

## Data Availability

The data presented in this study are available upon request from the first author Vivian Tullio. The data are not publicly available due to privacy concerns.
